# Early Sex Differences in pH, Base Excess and Lactate for the Prediction of Mortality in Trauma Patients

**DOI:** 10.3390/bioengineering13070799

**Published:** 2026-07-12

**Authors:** Philipp Vetter, Patrik Nothdurft, Cédric Niggli, Louisa Bell, Daniel Haschtmann, Hans-Christoph Pape, Ladislav Mica

**Affiliations:** 1 Department of Trauma Surgery, University Hospital Zurich, 8091 Zurich, Switzerland; 2 Centre for Trauma Surgery, 8027 Zurich, Switzerland; 3 Department of Spine Surgery, Schulthess Clinik, 8008 Zurich, Switzerland

**Keywords:** pH, base excess, lactate, mortality, trauma, sex

## Abstract

Background and Objectives: In trauma patients, early correction of acid–base imbalances is crucial for survival, but time-dependent and sex-specific predictive data are limited. The aim was to analyze the time-dependent, early sex differences in pH, base excess (BE) and lactate regarding mortality in trauma patients. Materials and Methods: Internal data of trauma patients served for retrospective analysis. pH, BE and lactate values were measured until 48 h after admission. Patients were compared according to sex and survivor status. pH < 7.25, BE < −5.5 mmol/L and lactate > 4 mmol/L were tested as time-dependent predictors for mortality, adjusted for age and Injury Severity Score (ISS). The respective area under the curve (AUC) was calculated and compared by sex. Level of significance was set at *p* < 0.05. Results: In the total cohort of 3653 patients (mean age: 45.8 ± 20.2 years, 73.4% male), mortality rates were comparable between females (26.7%) and males (26.9%; *p* = 0.931) at a similar ISS (median 25, respectively; *p* = 0.821). Female non-survivors had a higher rate and grade of pelvic injuries than male non-survivors (*p* = 0.005). At admission, males had more extreme values in pH (7.31 ± 0.13 vs. 7.32 ± 0.14; *p* < 0.001) and lactate (3.03 ± 2.53 vs. 2.71 ± 2.53; *p* < 0.001). pH and BE differed early by survivor status (within 4 h). Lactate was always higher in non-survivors. A pH < 7.25 was predictive in males at most time points (except for 0/4/8/48 h), but for females only at 12 h. BE < −5.5 mmol/L was predictive for males at 6 h and 12–48 h, and for females at 8–48 h. Lactate > 4 mmol/L was prognostic at 0–1 h and 6–48 h in males but in females at 0/12/24 h. Conclusions: Trauma patients display early differences in injury pattern and acid–base parameters according to survivor status and sex. Time- and sex-dependent referencing could aid in early risk estimation and the subsequent extent of surgical treatment.

## 1. Introduction

Trauma patients are at high risk of mortality [1,2,3]. Therefore, rapid and accurate assessment with early identification of those at risk is essential [4].

Previous outcome prediction implementations to forecast adverse events have reported the time-dependent role of pH, base excess (BE) and lactate [5,6,7,8,9,10,11,12], These parameters have been described as crucial and predictive for mortality in trauma patients [9,12,13,14,15,16,17].

Lactate has not only been interpreted in association with hypoperfusion but also with an adrenergic state [18], e.g., in trauma [19]. Its seemingly linear correlation with AE in the context of injury severity likely relates heterogenic cut-off values ranging from >2 mmol/L [14,20] to >4 mmol/L [9], thereby complicating decision-making. BE is often described with extreme threshold-values, but these limit specificity [16]. pH is a comprehensive parameter affected by oxygen debt, metabolic disbalance and generally by compensating mechanisms [21].

Despite threshold values (pH < 7.25, BE < −5.5 mmol/L and lactate > 4 mmol/L) for reducing complications [22], sex-specific studies on pH, BE and lactate for mortality lack consideration of the time aspect (values often limited to admission), sex, sample size and specificity [9,12,13,14,15,16,17,23,24,25]. One study, based on data from the same institution as the current study, found a predictive value for each acid–base parameter, particularly lactate, but the association with sex was not analyzed [15]. Opening the timeline in consideration of sex might lead to more precise insights about acid–base metabolism to enable early recognition, intervention, and correction.

The aim was to analyze the early sex differences in pH, BE and lactate for predicting mortality in trauma patients.

## 2. Materials and Methods

### 2.1. Ethical Approval

This study was conducted in accordance with the guidelines for good clinical practice and Helsinki as well as the TRIPOD (Transparent reporting of a multivariable prediction model for individual prognosis or diagnosis) statement [26]. Drafting of the document was based on the STROBE (Strengthening the Reporting of Observational Studies in Epidemiology) statement, a guideline used for observational studies [27]. Ethical approval was granted by the ethics commission of the University Hospital of Zurich and the local government of Zurich upon database implementation (Nr. StV: 1-2008, approved 31 January 2008), later reapproved (BASEC 2021-00391, approved 9 March 2021).

### 2.2. Patient Sample

This is a retrospective study conducted at a single center with data extraction from institutional database spanning from 1996 to 2022, including patients admitted to the trauma bay.

Exclusion criteria included patient death prior to admission and referred from external hospitals. Four groups were defined according to sex (male/female) and the occurrence of death (survivor/non-survivor) during hospital stay or within follow-up of up to three months. pH, BE and lactate were measured for each patient at time points (admission, 1, 2, 3, 4, 6, 8, 12, 24 and 48 h) after admission to our trauma bay [5,6,7,8].

### 2.3. Measuring Acid–Base Parameters

pH, BE [mmol/L] and lactate levels [mmol/L] were analyzed by use of a standardized blood gas analyzer at one institution (Institut für Klinische Chemie, University Hospital of Zurich).

### 2.4. Statistical Analysis

Data analysis was performed with SPSS 29.0 (IBM Corporation, Armonk, NY, USA). The baseline characteristics of the patient cohort are reported as means ± standard deviation for numerical variables. Ordinal data is presented as medians with interquartile ranges (IQR), while binary variables are described as percentages.

The cohort was initially divided by sex (male/female), which, in turn, were further divided by the occurrence of death (survivor/non-survivor). Group comparison was performed by sex and within each sex group by survivor status. Additionally, non-survivors of each sex were compared.

Binary logistic regression served for testing pH, BE and lactate as independent predictive factors for mortality. The analysis was adjusted for age and ISS, since those variables were previously identified as confounding factors in the context of trauma [8,12,13,14]. This was realized by adding the confounding variables into the regression equation. Goodness-of-fit was detected according to Nagelkerke R^2^ [28]. The parameters of interest were tested by group splitting according to previously described cut-off values of pH (<7.25), BE (<−5.5 mmol/L) and lactate (>4 mmol/L), originally reported as cut-offs for less pulmonary complications, which are, however, the most common at 8.2% in the original study [22] and 7% in the current study cohort and represent a common cause for “failure to rescue” [29]. Additionally, the odds ratio (OR) with 95% confidence interval (CI) was calculated in a 2 × 2 cross table based on the cut-off values (binary as yes or no) and mortality (binary as yes or no).

At each time point, the area under the curve (AUC) of pH, BE and lactate was calculated by sex; the two groups were compared according to Hanley and McNeil [30,31], and only groups with an AUC > 0.5 were considered for analysis. The level of significance was set a *p* < 0.05. Bonferroni correction was applied for multiple testing.

## 3. Results

In total, 3653 patients (mean age: 45.8 ± 20.2 years; 73.4% male) were included. Of these, 91.3% (n = 3336) were subject to blunt trauma. Non-survivors comprised 981 cases (26.9%). There were no differences in mortality according to sex, with a female rate of 26.7% (260/972) and a male rate of 26.9% (721/2681; *p* = 0.931).

The number of values included at each time point (with percentage of total cohort) are as follows: pH at admission: 2554 (70.0%); pH at 1 h: 1350 (37.0%); pH at 2 h: 1496 (41.0%); pH at 3 h: 1393 (38.1%); pH at 4 h: 1492 (40.8%); pH at 6 h; 1544 (42.3%); pH at 8 h: 1491 (40.8%); pH at 12 h: 1662 (45.5%); pH at 24 h: 1880 (51.5%); pH at 48 h: 1318 (36.1%); BE at admission: 2782 (76.2%); BE at 1 h: 1346 (36.8%); BE at 2 h: 1497 (41.0%); BE at 3 h: 1390 (38.1%); BE at 4 h: 1494 (40.9%); BE at 6 h: 1530 (41.9%); BE at 8 h: 1494 (40.9%); BE at 12 h: 1656 (45.3%); BE at 24 h: 1870 (51.2%); BE at 48 h: 1319 (36.1%); lactate at admission: 3044 (83.3%); lactate at 1 h: 1311 (35.9%); lactate at 2 h: 1425 (39.0%); lactate at 3 h: 1302 (35.6%); lactate at 4 h: 1423 (39.0%); lactate at 6 h: 1474 (40.4%); lactate at 8 h: 1435 (39.3%); lactate at 12 h: 1624 (44.5%); lactate at 24 h: 2105 (57.6%); lactate at 48 h: 1397 (38.2%).

Comparing females and males, which had a similar injury severity (ISS) score, females were older, had a higher Glasgow Coma Scale (GCS), a lower body temperature, systolic blood pressure (SBP), hemoglobin (HB) and body mass index (BMI) (Table 1). Injury-wise, there were differences for the abdomen, pelvis and spine, with females more often having such injuries (except for grade 5 injuries in males, according to the Abbreviated Injury Score, AIS). Males had more extreme values in pH and lactate.

### 3.1. Group Differences in Laboratory Parameters According to Survivor Status for Each Sex

The time-dependent values according to survivor status for each sex are presented in Table 2.

Both female and male non-survivors had a higher age and ISS, with a lower GCS, body temperature, prothrombin time and HB. Male non-survivors had a longer accident-to-admission interval. Comparing female and male non-survivors, female non-survivors were older and had a lower body temperature, HB and BMI.

Female non-survivors had more often severe injuries of the head and thorax (within AIS grade 4–6), but less often with injuries of the spine, extremities and integument. Similarly, male non-survivors presented more often with severe injuries to head, face, abdomen, extremities and integument (within AIS grade 4–6), but less often with injuries of the spine. Comparing non-survivors, females had a higher rate and grade of pelvic injuries.

### 3.2. Differences in pH, Base Excess and Lactate According to Survivor Status for Each Sex

**Figure 1:**
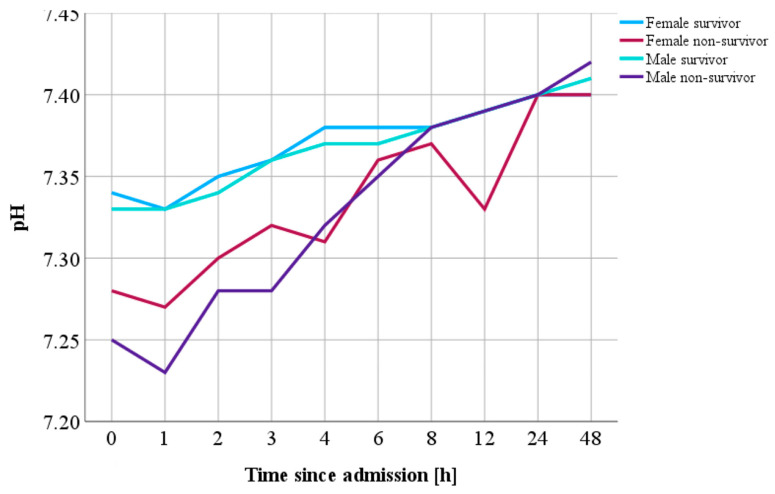
Differences in pH according to survivor status by sex.

The pH in female non-survivors was lower between 0 and 2 h (*p* < 0.001; *p* = 0.002 and *p* = 0.008, respectively) (Figure 1). For males, it was lower between 0 and 4 h (*p* < 0.001, respectively). Female non-survivors had lower values than their male counterparts at 12 h (*p* = 0.01).

**Figure 2:**
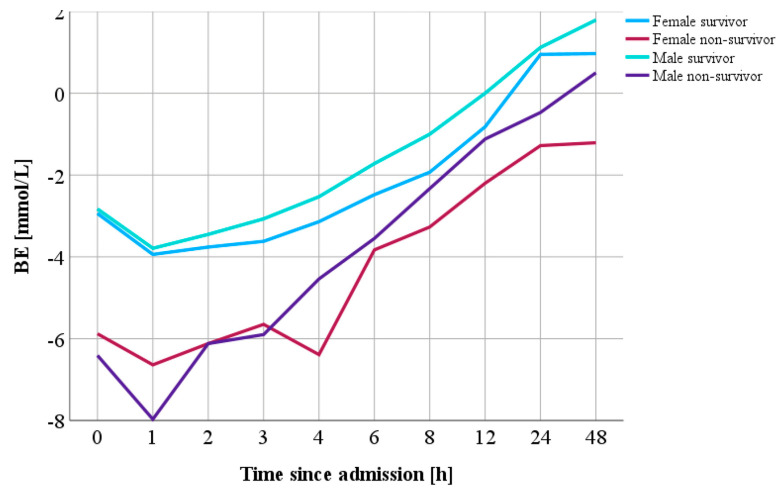
Differences in base excess according to survivor status by sex.

For BE in females, non-survivors had lower values between 0 and 1 h and at 4 h (*p* < 0.001; *p* < 0.001 and *p* = 0.001, respectively) (Figure 2), and between 8 and 48 h (*p* = 0.012; *p* = 0.009; *p* = 0.005 and *p* = 0.003, respectively). In males, there was a permanent difference throughout the measuring period (*p* < 0.001, respectively; at 12 h: *p* = 0.002).

**Figure 3:**
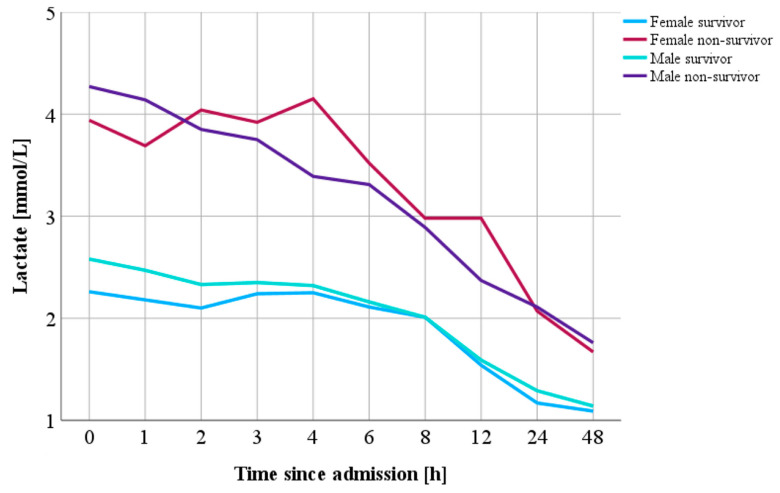
Differences in lactate according to survivor status by sex.

Regarding lactate, lactate was always higher in non-survivors for each sex group (females: *p* > 0.001, respectively; at 3/48 h: *p* = 0.001; at 8 h: *p* = 0.004) (males: *p* < 0.001, respectively) (Figure 3).

### 3.3. Parameters as Independent Predictors for Mortality

**Figure 4:**
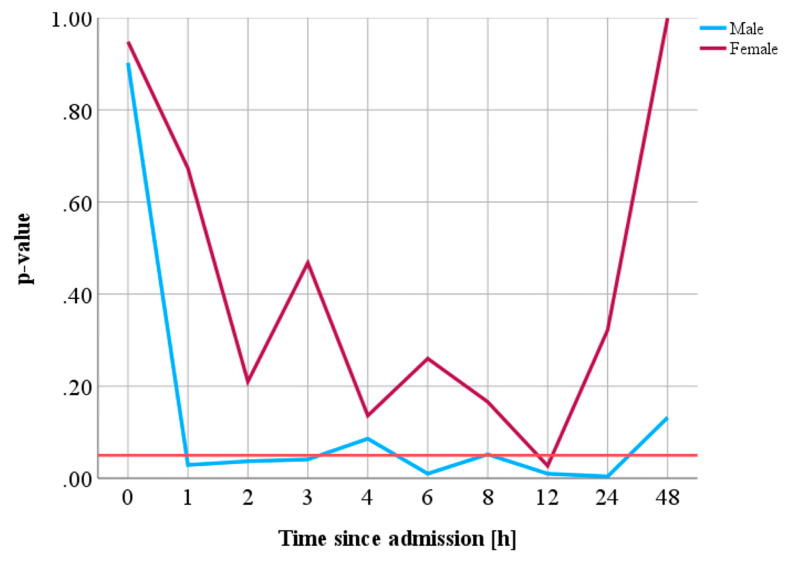
Analysis of pH being an independent predictive factor for mortality.

A pH < 7.25, adjusted for age and ISS, represented an independent predictive factor for mortality in males at most time points, except for 0/4/8/48 h (OR with 95% CI: 1 h: 2.69, 2.15–3.37; 2 h: 2.54, 2.06–3.13; 3 h: 2.83, 2.16–3.70; 6 h: 2.53, 1.73–3.70; 12 h: 4.30, 2.39–7.73 24 h: 4.43, 2.78–7.11), while it was only significant for females at 12 h (OR with 95% CI: 4.39, 2.09–9.21) (Figure 4).

**Figure 5:**
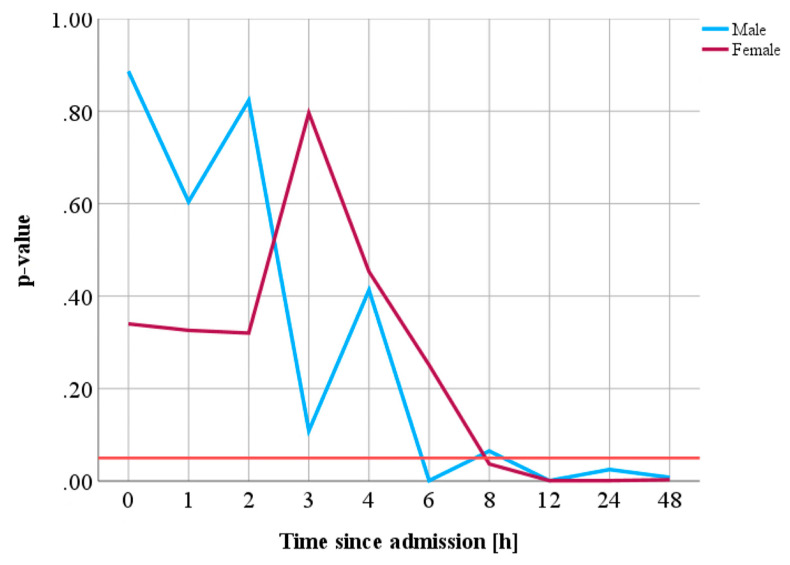
Analysis of base excess being an independent predictive factor for mortality.

Analogously, a BE of <−5.5 mmol/L was predictive for males at 6 h (OR with 95% CI: 2.44, 1.81–3.28) and 12–48 h (OR with 95% CI: 12 h: 2.97, 1.88–4.97; 24 h: 3.06, 1.85–5.08; 48 h: 6.36, 3.86–10.50), and for females at 8–48 h (Figure 5) (OR with 95% CI: 8 h: 2.18, 1.25–3.80; 12 h: 5.41, 3.28–8.93; 24 h: 4.90, 2.62–9.18; 48 h: 7.03, 3.70–13.37). 

**Figure 6:**
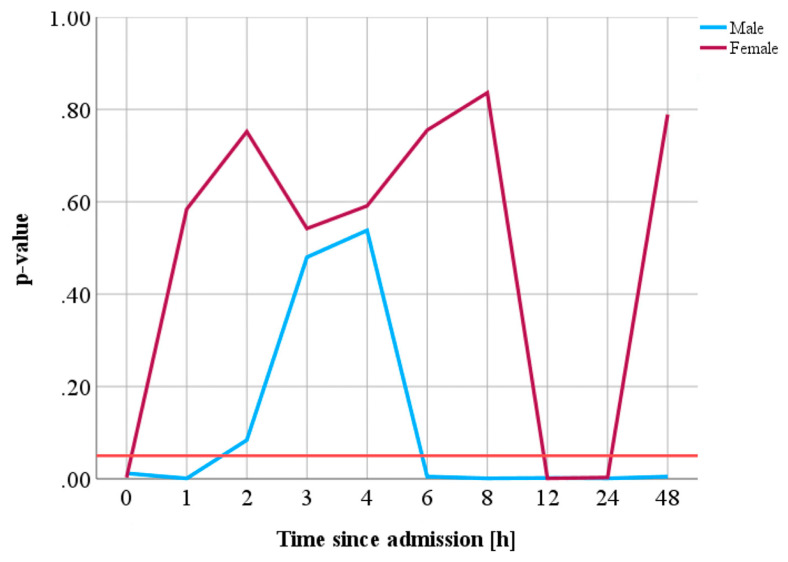
Analysis of lactate being an independent predictive factor for mortality.

A lactate value of >4 mmol/L in males was prognostic at 0–1 h (OR with 95% CI: 0 h: 2.30, 2.02–2.62; 1 h: 3.28, 2.37–4.54) and 6–48 h (OR with 95% CI: 6 h: 2.61, 1.94–3.51, 12 h: 3.16, 2.08–4.78; 24 h: 3.55, 2.60–4.84; 48 h: 3.97, 2.19–7.21) but in females at 0/12/24 h (OR with 95% CI: 0 h: 2.32, 1.86–2.90; 12 h: 5.48, 3.38–8.86; 24 h: 6.16, 3.86–9.86) (Figure 6).

Analysis was adjusted for age and Injury Severity Score. The level of significance (*p* = 0.05) is depicted by the horizontal light red line.

Goodness-of-fit values are reported in Table 3.

### 3.4. Area Under the Curve

**Figure 7:**
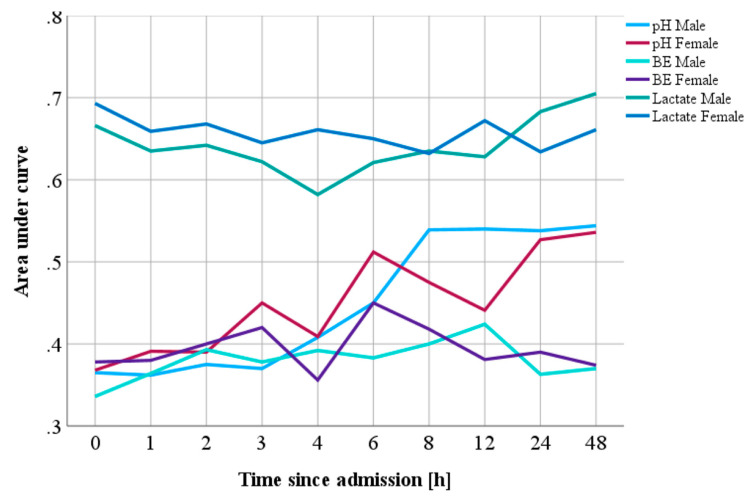
Analysis of pH, base excess and lactate regarding area under the curve.

Lactate always had an AUC > 0.5 (Figure 7). The AUC of pH increased to > 0.5 after 6 h, 24 h and 48 h in females or from 8 h onwards in males, while BE always remained below this threshold. For values with an AUC > 0.5, there were no differences between sexes at any time point (*p* > 0.05).

## 4. Discussion

This study corroborates the relevance of sex differences and pH, BE and lactate for mortality in trauma patients [15,16]. It shows that group differences according to survivor status in acid–base parameters exist very early, and that parameters represent significant independent predictive factors for mortality. Lactate had the highest predictive value at each time point.

Age and ISS are known confounding variables in trauma [8,12,14,25,32,33,34]. This notion is re-affirmed by the current results: There were very early differences at consecutive time points for pH, BE and lactate between survivors and non-survivors. However, non-survivors had a longer accident-to-admission-interval, higher age and Injury Severity Score (ISS), and a lower GCS, body temperature, SBP, prothrombin time, HB, and different injury patterns, which likely explains that only few time-related values represented independent predictors for mortality.

The current study implies that acid–base parameters differ within the first few hours; implying that an early time frame of a few hours after admission is essential for avoiding mortality and should be recognized as such. It must also be stressed that even slightly deranged acid–base parameters are significant in their association with mortality, urging physicians to be aware of such subtle signs.

The main advantage of this study is the referencing of closely monitored laboratory parameters that not only provide information during initial assessment but also enable re-evaluation over the further course of hospital stay, as patients may still require major treatment despite having been hemodynamically stabilized.

Following up on a previous study connecting pH, BE and lactate with mortality in trauma patients [15], we provided a sex-specific analysis. Here, we observed pH and lactate as more constant predictors for mortality in males.

As previously explained [35,36], sex differences have been mainly explained by differences in hormonal and immunological status [37,38], such as estrogen (particularly before menopause [24]) being a protective factor. On an immunological level, estrogen provides an anti-inflammatory effect by regulating immune cells and amplifying the production of anti-inflammatory cytokines (for example, interleukin, IL-10) while reducing pro-inflammatory cytokines (for example, tumor necrose factor alpha and IL-6) [39,40].

Schoeneberg et al. reported less abnormal values in BE and lactate in females and stated that lactate as a predictor for mortality could not be validated in female trauma patients [25]. Our time-dependent analysis provides a more nuanced result, finding a predictive value for lactate at admission and at 12/24 h.

Brauckmann et al. evaluated 80 patients with a mean age of 45.6 years and a mean ISS of 35.4 (treated at the intensive care unit, ICU), with a mortality rate of 14% and a higher ISS in non-survivors (49) [23]. Females were slightly older, had a lower BMI, and had more severe head and neck injuries at a tendentially higher ISS (41 vs. 34). There were no differences in mortality in the small cohort. Given the difference in only head and neck injuries (suggesting mainly traumatic brain injuries), it is questionable if this cohort is representative.

Trentzsch et al. matched pairs (n = 3887) of females and males by age, mechanism, AIS (head, thorax, abdomen and extremities) and occurrence of prehospital shock [24]. Their cohort was slightly younger (42.5 years) but had comparable values in ISS (approx. 24.8). BE at admission and lactate at ICU admission revolved around −3 and 3 mmol/L, respectively. Mortality was higher in males (17.8%) than in females (16.1%).

The current study provides a comprehensive view on injury patterns, including pelvis, spine and integument. Of note, females more often had injuries to the abdomen, pelvis and spine, and female non-survivors less often had injuries of the spine and integument. Comparing non-survivors, females had a higher rate and grade of pelvic injuries. Abt et al. analyzed the three previously mentioned acid–base parameters in trauma patients with severe pelvic injuries and found early differences between survivors and non-survivors, suggesting BE as the strongest discriminative parameter [16]. Our results provide a differentiated perspective on injury patterns in female patients, particularly enforcing heightened awareness for pelvic injuries that could trigger acid–base imbalances.

Several limitations must be accounted for: The association of pH, BE and lactate with mortality in trauma patients is well known [9,12,13,14,15,16,17] and has steadily expanded. There were changes in treatment protocols (especially fluid and hemostatic resuscitation efforts leading to dilution, yet more restrictive regarding crystalloids and an administration of blood and plasma [41]) over the observational period of 26 years.

The time frame of measuring the laboratory parameters was rather short and is due to the isolated measuring at our ICU. Missing values were not replaced, and the number of available measurements was lower over time. This introduces a selection bias, although it represents daily habits to some extent. This may also explain the tendency of increasing odds for mortality with increasing time after admission. The medical history of the patient (including comorbidities, such as renal insufficiency) was not considered. This study’s retrospective design may introduce potential biases and confounding factors that could affect the observed associations. Furthermore, the institution represents a Level I Trauma Center, which limits comparison to other (more regional) hospitals.

## 5. Conclusions

Trauma patients display early differences in injury pattern and acid–base parameters according to survivor status and sex. Time- and sex-dependent referencing could aid in early risk estimation and the subsequent extent of surgical treatment.

## Figures and Tables

**Table 1:** Parameters on admission day between females and males. AIS, Abbreviated Injury Score; Accident-to-admission-interval [min]; BE, base excess [mmol/L]; BMI, Body Mass Index [kg/m^2^]; CRP, C-reactive Protein [mg/L]; GCS Glasgow Coma Scale; HB, Hemoglobin [g/dL]; ISS, Injury Severity Score; lactate [mmol/L]; PT, Prothrombin Time [% of reagence]; SBP, systolic blood pressure [mmHg]; body temperature [°C].

Parameter	Female (n = 972)	Male (n = 2681)	*p*-Value
Accident-to-admission-interval	63.7 ± 34.3 ^1^	64.8 ± 34.0 ^1^	0.306
Age	50.9 ± 21.7 ^1^	43.9 ± 19.2 ^1^	<0.001
ISS	25 (17–34) ^2^	25 (17–34) ^2^	0.821
AIS (0–6)			
-Head	216/72/76/149/207/221/28 ^3^	633/187/200/405/512/646/81 ^3^	0.775
-Face	705/64/116/61/6/0/0 ^3^	1913/153/353/159/51/2/0 ^3^	0.066
-Thorax	466/46/53/268/87/40/2 ^3^	1258/99/134/771/272/121/2/0 ^3^	0.524
-Abdomen	630/6/93/71/89/64/0 ^3^	1916/18/144/172/241/138/1 ^3^	<0.001
-Pelvis	680/11/75/147/31/9/0 ^3^	2180/15/99/248/51/18/0 ^3^	<0.001
-Spine	657/4/136/117/19/21/0 ^3^	1886/22/321/260/43/88/6 ^3^	0.027
-Extremities	438/63/208/186/53/12/0 ^3^	1273/117/556/506/137/52/0 ^3^	0.083
-Integument	586/248/97/14/2/0/0 ^3^	1637/624/286/43/12/11/0 ^3^	0.214
GCS	11 (3–15) ^2^	8 (3–15) ^2^	0.014
Body temperature	35.3 ± 1.9 ^1^	35.6 ± 1.6 ^1^	<0.001
SBP	130 ± 28 ^1^	131 ± 27 ^1^	0.037
PT	85 (65–99) ^2^	84 (65–97) ^2^	0.197
HB	10.5 ± 5.1 ^1^	11.7 ± 3.5 ^1^	<0.001
CRP	13.0 ± 39.3 ^1^	14.0 ± 42.2 ^1^	0.847
BMI	23 (21–26) ^2^	25 (23–27) ^2^	<0.001
pH	7.32 ± 0.14	7.31 ± 0.13	<0.001
BE	−3.74 ± 5.46	−3.79 ± 5.20	0.630
Lactate	2.71 ± 2.53	3.03 ± 2.53	<0.001

^1^: Mean with standard deviation; ^2^: median with interquartile ranges; ^3^: absolute numbers.

**Table 2:** Parameters on admission day between survivors and non-survivors. AIS, Abbreviated Injury Score; Accident-to-admission-interval [min]; BE, base excess [mmol/L]; BMI, Body Mass Index [kg/m^2^]; CRP, C-reactive Protein [mg/L]; GCS Glasgow Coma Scale; HB, Hemoglobin [g/dL]; ISS, Injury Severity Score; lactate [mmol/L]; PT, Prothrombin Time [% of reagence]; SBP, systolic blood pressure [mmHg]; body temperature [°C].

Parameter	Female Survivors (n = 712)	Female Non-Survivors (n = 260)	*p*-Value	Male Survivors (n = 1960)	Male Non-Survivors (n = 628)	*p*-Value	*p*-Value (Female/ Male Non-Survivors)
Accident-to-admission-interval	62.6 ± 35.2 ^1^	66.7 ± 31.6 ^1^	0.036	63.2 ± 34.6 ^1^	69.8 ± 32.0 ^1^	<0.001	0.352
Age	47.9 ± 20.3 ^1^	59.1 ± 23.1 ^1^	<0.001	41.8 ± 17.8 ^1^	49.8 ± 21.7 ^1^	<0.001	<0.001
ISS	22 (16–30) ^2^	34 (25–50) ^2^	<0.001	25 (16–34) ^2^	33 (25–45) ^2^	<0.001	0.330
AIS (0–6)							
-Head	192/71/68/132/152/95/0 ^3^	24/1/8/17/55/126/28 ^3^	<0.001	553/177/185/344/384/301/3 ^3^	80/10/15/61/128/345/78 ^3^	<0.001	0.514
-Face	509/53/91/43/4/0/0 ^3^	196/11/25/18/2/0 ^3^	0.253	1355/125/282/128/34/1/0 ^3^	558/28/71/31/17/1/0 ^3^	<0.001	0.341
-Thorax	342/37/44/201/58/23/0 ^3^	124/9/9/67/29/1 ^3^	0.01	882/82/114/591/198/82/0 ^3^	376/17/20/180/74/39/2 ^3^	<0.001	0.722
-Abdomen	448/5/79/58/70/39/0 ^3^	182/1/14/13/19/25/0 ^3^	0.04	1376/14/122/147/181/85/0 ^3^	540/4/22/25/60/53/1 ^3^	<0.001	0.363
-Pelvis	497/7/54/113/24/3/0 ^3^	183/4/21/34/7/6/0 ^3^	0.103	1598/11/68/190/37/8/0 ^3^	582/4/31/58/14/10/0 ^3^	0.072	0.005
-Spine	457/4/111/94/17/16/0 ^3^	200/0/25/23/2/5/0 ^3^	0.005	1340/20/241/35/69/2 ^3^	546/2/80/46/8/19/4 ^3^	<0.001	0.563
-Extremities	290/52/167/152/40/6/0 ^3^	148/11/41/34/13/6/0 ^3^	<0.001	847/98/431/411/116/35/0 ^3^	426/19/125/95/21/17/0 ^3^	<0.001	0.489
-Integument	408/200/78/10/1/0/0 ^3^	178/48/19/4/1/0/0 ^3^	0.007	1154/492/227/26/7/7/0 ^3^	483/132/59/17/5/4/0 ^3^	<0.001	0.764
GCS	14 (3–15) ^2^	3 (3–4) ^2^	<0.001	14 (3–15) ^2^	3 (3–3) ^2^	<0.001	0.283
Body temperature	35.5 ± 1.5 ^1^	34.5 ± 2.7 ^1^	<0.001	35.9 ± 1.3 ^1^	35.0 ± 2.2 ^1^	<0.001	0.006
SBP	131 ± 25 ^1^	124 ± 37 ^1^	0.026	133 ± 25 ^1^	122 ± 34 ^1^	<0.001	0.804
PT	90 (73–100) ^2^	65 (42–84) ^2^	<0.001	86 (71–98) ^2^	71 (49–89) ^2^	<0.001	0.067
HB	11.0 ± 5.6 ^1^	9.4 ± 3.1 ^1^	<0.001	12.1 ± 2.7 ^1^	10.7 ± 5.1 ^1^	<0.001	<0.001
CRP	13.5 ± 38.8 ^1^	11.2 ± 36.6 ^1^	0.946	15.0 ± 44.4 ^1^	10.9 ± 34.5 ^1^	0.134	0.532
BMI	23 (21–26) ^2^	23 (21–25) ^2^	0.263	25 (23–27) ^2^	26 (24–28) ^2^	0.154	<0.001

^1^: Mean with standard deviation; ^2^: median with interquartile ranges; ^3^: absolute numbers.

**Table 3:** Goodness-of-fit for mortality. BE, base excess [mmol/L]; lactate [mmol/L].

Time Since Admission [h]	pH Female	pH Male	BE Female	BE Male	Lactate Female	La Male
0	0.491	0.428	0.514	0.437	0.489	0.443
1	0.443	0.316	0.450	0.305	0.503	0.402
2	0.428	0.308	0.397	0.284	0.413	0.309
3	0.422	0.256	0.411	0.256	0.441	0.275
4	0.393	0.246	0.400	0.242	0.444	0.276
6	0.343	0.209	0.342	0.216	0.404	0.251
8	0.320	0.214	0.327	0.213	0.375	0.245
12	0.267	0.191	0.309	0.196	0.353	0.220
24	0.193	0.298	0.223	0.193	0.315	0.261
48	0.192	0.141	0.241	0.150	0.229	0.148

## Data Availability

Production data is available upon reasonable request.

## Abbreviations

The following abbreviations are used in this manuscript:

AIS	Abbreviated injury score
AUC	Area under the curve
BE	Base excess
BMI	Body mass index
CRP	C-reactive protein
GCS	Glasgow Coma Scale
HB	Hemoglobin
ICU	Intensive care unit
IQR	Interquartile range
ISS	Injury severity score
PT	Prothrombin time
SBP	Systolic blood pressure
STROBE	Strengthening the Reporting of Observational Studies in Epidemiology
TRIPOD	Transparent reporting of a multivariable prediction model for individual prognosis or diagnosis
